# Practical Notes on Diagnosis and Treatment

**Published:** 1907-07-13

**Authors:** 


### PRACTICAL NOTES ON DIAGNOSIS AND TREATMENT.

**Dry Eczema with Pruritus.**

In this condition such an ointment as the following is useful: Menthol, gr. xxx.; resorcin, gr. xv.; precipitated sulphur, ʒij.; zinc oxide, ʒiij.; vaseline, ℥j.

**Tobacco Amblyopia.**

Patients suffering from tobacco poisoning often notice that they have difficulty in distinguishing between gold and silver coins. Another common statement is that they see better in the dusk of the evening than in the bright light or sunshine.

**Cramps in Diabetes.**

Cramps in the lower limbs are common in cases of diabetes. They are rare in the daytime, but may be very troublesome at night. They occur principally in the calves, but also affect other leg muscles. *Dr. Benjamin Ward Richardson.*

**Vaginal Discharges.**

When the acute inflammatory condition of the vaginal mucous membrane has subsided, or where it does not exist, and when the exudation has become well established, the local application of ichthyol in the form of a 10 per cent. ointment may prove remarkably effectual.—*Dr. More-Madden.*

**Psoriasis.**

When this affects a considerable area the patient may be given a sulphur bath for three-quarters of an hour, during which the patches should be vigorously rubbed with soap. Then apply to the affected areas a mixture containing oil of cade 2 drachms, collodion 5 drachms.

**Laryngeal Irritation.**

The following formula, taken from the Brompton Pharmacopœia, is often useful in allaying laryngeal irritation: Glycerine of carbolic acid ʒj., chloroform ♏ x., added to a pint of boiling water and the steam inhaled. In some cases relief is obtained by sucking ice.

**Chloasma of Pregnancy.**

Dr. Hare recommends the following: Zinci oxid., grs. vj.; hydrarg. ammon., grs. ij.; ol. theobrom, ʒv.; ol. ricini, ʒv.; ol. rosæ, ♏ x. Apply to the face night and morning.

**Optic Neuritis in Chlorosis.**

The most intense optic neuritis, precisely like that of cerebral tumour, may be due to anæmia, at any rate to the chlorotic anæmia of girls.—*Sir William Gowers.*

**Impacted Cerumen.**

When the cerumen is hard, dry, and adherent, use a solution of sodium carbonate (10 grains) in 2 drachms each of glycerine and water. Introduce a few drops into the ear several times a day, and then in the course of a few days the wax will be softened and can be removed by injections of warm water. Afterwards put a plug of cotton-wool in the meatus and leave it for 24 hours.

**Phthisis Pulmonalis.**

When with active mischief in one lung the other lung becomes infected, the first signs of this accident will not be found in the extreme apex of the secondarily affected lung, but in other situations—namely, in the case of the right lung by examining over the second right costal cartilage near the sternum, and in the case of the left in the anterior axillary line just above the level of the nipple.—*Dr. J. Edward Squire.*

**Parametritis and Albuminuria.**

Matthews Duncan showed that in parametritis albuminuria often occurred. This is sometimes due to bursting of an abscess into the bladder or to the coincident existence of cystitis or of Bright's disease. But apart from these cases there are others in which there is, during parametritis, a considerable amount of albumen in the urine—half its bulk or more without either casts or pus. As the parametritis gets well, so does the albuminuria.—*Dr. Herman.*

